# Prevalence and determinants of caesarean section deliveries in the Kintampo Districts of Ghana

**DOI:** 10.1186/s12884-023-05622-5

**Published:** 2023-04-25

**Authors:** Daniel Gyaase, Yeetey Akpe Enuameh, Benjamin Noble Adjei, Stephaney Gyaase, Emmanuel Kweku Nakua, Moses Musah Kabanunye, Mohammed Muhib Alhassan, Mohammed Sheriff Yakubu, Richard Joshua Tetteh, Sam Newton, Kwaku Poku Asante

**Affiliations:** 1 Department of Epidemiology and Biostatistics, School of Public Health, Kwame Nkrumah University of Science and Technology, Kumasi, Ghana; 2 Kintampo Health Research Centre, Research and Development Division, Ghana Health Service, Kintampo, Ghana; 3 London School of Hygiene and Tropical Medicine, Keppel St, London, WC1E 7HT United Kingdom; 4 Department of Global and International Health, School of Public Health, Kwame Nkrumah University of Science and Technology, Kumasi, Ghana

**Keywords:** Caesarean section, Prevalence, Determinants, Obstetric

## Abstract

**Background:**

Globally, the increasing rate of caesarean section (CS) delivery has become a major public health concern due to its cost, maternal, neonatal, and perinatal risks. In Ghana, the Family Health Division of the Ghana Health Service in 2016 opted to initiate a program to prevent the abuse of CS and identify the factors contributing to its increase in the country. This study aimed to determine the prevalence and factors influencing CS deliveries in the Kintampo Districts of Ghana.

**Methods:**

The current study used secondary data from the Every Newborn–International Network for the Demographic Evaluation of Populations and their Health (EN-INDEPTH) project in Kintampo, Ghana. The outcome variable for this study is CS delivery. The predictor variables were socio-demographic and obstetric factors.

**Results:**

The prevalence of CS delivery in the study area was 14.6%. Women with secondary education were 2.6 times more likely to give birth by CS than those with primary education. Unmarried women were about 2.5 times more likely to deliver by CS compared to those who were married. There was an increasing order of CS delivery among women in the wealthy quintiles from poorer to richest. The likelihood of women with gestational ages from 37 to 40 weeks to give birth by CS was about 58% less compared to those with less than 37 gestational weeks. Women who had 4–7 and 8 or more antenatal care (ANC) visits were 1.95 and 3.5 times more likely to deliver by CS compared to those who had less than 4 ANC visits. The odds of women who have had pregnancy loss before to deliver by CS was 68% higher compared to women who have not lost pregnancy before.

**Conclusions:**

Caesarean section delivery prevalence in the study population was within the Ghana Health Service and World Health Organization ranges. In addition to known socio-demographic and obstetric factors, this study observed that a history of pregnancy loss increased the chances of a woman undergoing a CS. Policies should aim at addressing identified modifiable factors to stem the rise in CS deliveries.

## Background

The rate of caesarean section (CS) delivery has increased over the years across the globe [1, 2]. According to the World Health Organization (WHO), more than 21% and 9.2% of childbirths globally and in Africa respectively are from CS [3, 4]. Though there is a coexistence of unmet needs and overuse of the procedure, CS rate is projected to increase to 29% by 2030 [4]. The increase has become a major concern in public health due to its associated maternal, neonatal and perinatal risks, cost, and inequity of access [3, 5]. Without effective interventions to combat this trend, maternal morbidity and mortality will increase due to the unmet need and overuse of the procedure [4]. The procedure is performed when vaginal delivery poses a risk to a woman or baby’s life [6]. The decision to perform CS is based on the best way to save the lives of the mother and child [7].

In 2014, WHO conducted a systematic review and ecological analysis of CS rates for regions due to the concern by governments and clinicians to revisit the earlier (1985) recommended rates of 10 − 15% for all regions [6]. The review concluded that CS rates between 10 and 15% were associated with decreased maternal, neonatal and infant mortality, but higher CS rates did not demonstrate associations with mortality reduction [6, 8, 9]. In spite of the above evidence, countries’ CS rates keep increasing [4, 8]. Like all other surgeries, there are risks associated with CS. Unnecessary CS could increase maternal, neonatal, and infant morbidity and mortality [10]. A study by Verma and colleagues in South and South-East Asia reported that women in high and low-to-middle income countries now consider CS a preferred mode of delivery over vaginal [11]. The high cost of CS increases families’ health expenditure and puts extra pressure on the weak health systems, particularly in low-to-middle income countries [12].

Factors other than obstetric indications, such as anxiety about vaginal delivery, mother’s preference, educational level, and economic status, among others, have been observed to influence the performance of CS [1, 13]. In Ghana, the rate of CS deliveries increased from 14.6% in 2015 to 16.0% in 2016, a year after the WHO review. All administrative regions of Ghana except the Upper East Region recorded an increase in CS deliveries in the said year [14]. The Family Health Division of Ghana Health Service (GHS) 2016, aiming to prevent the abuse of the procedure, advocated the investigations of factors contributing to the continued upward trend in CS rates and examined the impact of the increasing rate on outcomes of deliveries in the country [14]. To the best of our knowledge, this is the first study to estimate CS prevalence and its determinants in the Kintampo Districts in the Bono East Region of Ghana. The current study seeks to complement GHS efforts to investigate the drivers of CS deliveries in the country.

The study aims to determine the prevalence and factors contributing to CS in the study area of Kintampo Health and Demographic Surveillance System (KHDSS), in the Kintampo North Municipality and Kintampo South District of Ghana.

## Methods

### Data source and description

The study used secondary data from Every Newborn–International Network for the Demographic Evaluation of Populations and their Health (EN-INDEPTH) study at Kintampo North Municipality and Kintampo South District in the Bono East Region of Ghana. This was a cross-sectional study conducted in communities served by the KHDSS from July 2017 to August 2018. The purpose of the original study was to compare two methods of recording pregnancy outcomes, the Full Birth History (FBH+) and Full Pregnancy History (FPH) methods, with random allocation at the individual woman level. Details of the study, including the selection of sites and sample size estimation, have previously been published [15].

### Study setting

Communities under the coverage of the KHDSS of the Kintampo Health Research Center (KHRC) contributed data to this study.

### Study population and sample size

The study population was women who have ever given birth and responded to the question on CS delivery in the questionnaire. Out of 12,194 women who took part in the survey, a sample of 2,887 responded to the CS delivery question. Using the STATA 16 statistical software, a significance level of 0.05 and a CS prevalence of 13.4% in the then Brong Ahafo Region of Ghana [14], a sample size of 2,887 provides a power of 99.9% to address the study objective.

### Study variables

The outcome variable for this study was CS delivery, which was measured as “Was (NAME) delivered by caesarean, that is, did they cut your belly open to take the baby out?”

The main predictors were obstetric characteristics (such as parity, Antenatal care [ANC] visits, gestational age, place of birth, and mother’s perceived baby size at birth.) and socio-demographic characteristics (maternal age, level of education, religion, ethnicity, marital status, and wealth quintiles.). Gestational age was measured as “how many weeks pregnant were you when baby was born”. Based on the number of weeks, gestational age was categorised as < 37 weeks (preterm), 37–40 weeks (term), and above 40 weeks (post-term). Mother’s perceived baby size at birth was categorized as large, average, and small. Other covariates, such as pregnancy intentions, and previous pregnancy loss, were assessed for their influence on CS delivery. The wealth quintiles originally constructed for the total sample of 12,194 were derived from socioeconomic indicators such as assets owned, housing, and infrastructure of households using principal component analysis [16]. The current study used a sub-set of the original sample, hence the wealth quintiles not being evenly distributed (20% ±1).

### Statistical analysis

Data were summarised using frequencies and percentages for all variables. Univariable and multivariable logistic regression models were used to assess factors influencing CS delivery. Independently significant variables (≤ 0.05) in the univariate analysis and variables considered of biological importance (pregnancy intentions, family planning use, etc.) but were not significant in the univariate analysis were included in the multivariable logistic regression model. The automated stepwise approach was used in adding variables to the multivariable logistic regression model. The model’s goodness of fit was assessed using the Akaike information criterion (AIC). The model with the smallest AIC value was considered the best fit. Confidence intervals were computed at a 95% confidence level, and a p-value < 0.05 was considered statistically significant for the final model. Data were analysed using STATA version 16.0 (Stata Corp, College Station, USA).

## Results

### Socio-demographic characteristics of study respondents

Table 1 presents the socio-demographic characteristics of the respondents in relation to their CS deliveries. A total of 2,887 women aged (15–49 years) that had ever given birth were included in the analysis. The mean age of the women was 34 years, with a standard deviation of 7.02. Most women were aged 35 years and above (52.5%), followed by those between 25 and 34 years (37.9%). About 60% of the women had formal education, with a higher proportion (52.7%) having attained primary and a few (about 2.0%) tertiary education. Christianity and Dagomba were the respondents’ predominant religion and tribe (ethnicity) (43.4% and 39.3%, respectively). Up to 94.0% of the women were married. Most (28.3%) women were in the richer category of the wealth quintile, and the least (9.8%) were in the poorest wealth quintile.

The prevalence of CS delivery was 14.6% (421/2887, 95% CI 13.34–15.92). Women who delivered by CS had the following characteristics: 64.1% were above 35 years, and about 65.0% had formal education. Of those who had formal education, 53.2% attained primary education. Half (50.1%) of the women who gave birth by CS were Christians. A higher proportion (30.2%) of the women who delivered through CS belong to the richer wealth quintile category (Table 1).

**Table 1:** Socio-demographic characteristics of study participants by caesarean section deliveries

Variables	Caesarean section		
	**No (n = 2,466)** n (%)	**Yes (n = 421)** n (%)	**Total (n = 2887)** n (%)
**Maternal age**			
15-24 years	237 (9.6)	18 (4.3)	255 (8.8)
25–34 years	934 (37.9)	133 (31.6)	1,067 (37.0)
≥ 35 years	1,295 (52.5)	270(64.1)	1,565 (54.2)
**Level of education**			
No Education	1,001 (40.6)	149 (35.4)	1,150 (39.8)
Primary	1,296 (52.6)	224 (53.2)	1,520 (52.7)
Secondary	120 (4.8)	41 (9.7)	161 (5.5)
Higher	49 (2.0)	7 (1.7)	56 (2.0)
**Religion**			
Christian	1,042 (42.3)	211 (50.1)	1,253 (43.4)
Muslim	843 (34.2)	142 (33.7)	985 (34.1)
No religion	102 (4.1)	11 (2.6)	113 (3.9)
Other religion	479 (19.4)	57 (13.6)	536 (18.6)
**Ethnicity**			
Akan	588 (23.8)	120 (28.5)	708 (24.5)
Frafra	156 (6.3)	26 (6.2)	182 (6.3)
Waala	500 (20.3)	57 (13.5)	557 (19.3)
Dagomba	940 (38.1)	195 (46.3)	1,135 (39.3)
Fulani	110 (4.5)	10 (2.4)	120 (4.2)
Other	172(7.0)	13 (3.1)	185 (6.4)
**Marital Status**			
Married	2,317 (94.0)	380 (90.3)	2,697 (93.4)
Not married	149 (6.0)	41 (9.7)	190 (6.6)
**Wealth quintiles**			
Poorest	241 (9.8)	20 (4.8)	261 (9.0)
Poorer	475 (19.3)	71 (16.9)	546 (18.9)
Middle	548 (22.2)	110 (26.1)	658 (22.8)
Richer	698 (28.3)	127 (30.1)	825 (28.6)
Richest	504 (20.4)	93 (22.1)	597 (20.7)

### Obstetric characteristics of study participants

Table 2 presents the obstetric characteristics of the participants in the study. Up to 15.2% of women who gave birth through CS had more than 8 ANC visits. An overwhelming proportion (86.7%) of those who delivered through CS has had three or more births. A majority (86.4%) of CS deliveries occurred within the normal gestational ages (37–40 weeks). Most CS deliveries (65.0%) were done within hospitals.

Most CS deliveries (57.4%) were performed for mothers with babies of large body size.

**Table 2:** Obstetric characteristics of study participants by caesarean section deliveries

Variables	Caesarean section		
	No (n = 2,466) n (%)	Yes (n = 421) n (%)	Total (n = 2887) n (%)
**Number of ANC visits**			
< 4 visits	1,230 (49.9)	199 (47.3)	1,429 (49.5)
4–7 visits	1,015 (41.2)	158 (37.5)	1,173 (40.6)
8 and more Visits	221 (8.9)	64 (15.2)	285 (9.9)
**Parity**			
1 birth	176 (7.1)	10 (2.4)	186 (6.4)
2 births	247 (10.0)	46 (10.9)	293 (10.2)
≥ 3 births	2,043 (82.9)	365 (86.7)	2,408 (83.4)
**Gestational age (n = 2647)**			
Normal	2,101 (92.5)	325 (86.4)	2,426 (91.7)
Preterm	36 (1.6)	16 (4.3)	52 (2.0)
Post term	134 (5.9)	35 (9.3)	169 (6.4)
**Place of birth (n = 2380)**			
Hospital	990 (47.7)	197 (65.0)	1,187 (49.9)
Health centre/clinic	499 (24.0)	63 (20.8)	562 (23.6)
Health post	21 (1.0)	0 (0.00)	21 (0.9)
Private hospital/clinic	567 (27.3)	43 (14.2)	610 (25.6)
**Perceived Baby Size at birth (n = 2393)**			
Large	1,073 (51.4)	174 (57.4)	1,250 (52.2)
Average	829 (39.7)	92 (30.4)	921 (38.5)
Small	185 (8.9)	37 (12.2)	222 (9.3)

ANC = Antenatal care

### Factors influencing caesarean section deliveries

Table 3 presents the crude and adjusted logistic regression of obstetric and socio-demographic factors associated with CS delivery in the study area.

The crude and adjusted analysis showed significant associations between the eight (8) independent variables and CS delivery. The variables were maternal age, educational level, marital status, wealth quintiles, number of ANC visits, parity, gestational age, and previous pregnancy loss.

After controlling for other variables at a 95% confidence level, women aged 35 years and above (aOR = 2.93, 95%CI: 1.607, 5.361) and 25–35 years (aOR = 1.91, 95%CI: 1.073, 3.435) were 2.93 and 1.91 times more likely to deliver by CS compared to women aged 15–24 years respectively.

Women with secondary education (aOR = 2.71, 95%CI: 1.737, 4.235) were 2.71 times more likely to give birth by CS than those with primary education.

Unmarried women (aOR = 2.31, 95%CI: 1.552, 3.444) were about 2.31 times more likely to deliver by CS compared to those who were married.

Women were more likely to deliver by CS in the poorer (aOR = 1.83, 95%CI: 1.075, 3.103), middle (aOR = 2.00, 95%CI: 1.193, 3.347) and richer (aOR = 1.87, 95%CI: 1.121, 3.114) wealth quintiles compared to those in the poorest quintile.

Women who had 8 and more (aOR = 2.05, 95%CI: 1.467, 2.861) ANC visits were 2.05 times more likely to deliver by CS compared to those who had less than 4 ANC visits.

Women with two births (aOR = 4.26, 95%CI: 2.017, 9.020) and those with three or more births (aOR = 3.26, 95%CI: 1.560, 6.822) were about 4 and 3 times respectively more likely to have a CS delivery compared to those with one birth.

The likelihood of women delivering by CS is about three times higher during preterm compared to women delivering during term (aOR = 3.402, 95%CI: 0.156, 0.553), and those who deliver post-term are 50% higher to deliver by CS compared to those who deliver at term (aOR = 1.502, 95%CI: 0.998, 2.259).

The odds of women with previous pregnancy loss (aOR = 2.03, 95%CI: 1.467, 2.575) to deliver by CS was 2.03 times higher compared to women without previous pregnancy.

**Table 3:** Obstetric and socio-demographic factors influencing caesarean section delivery

Variables	Crude		Adjusted	
	**cOR**	**95%CI**	**aOR**	**95%CI**
**Maternal age**				
15-24 years	1		1	
25–34 years	1.87	1.123, 3.130	1.92	1.073, 3.435
≥ 35 years	2.75	1.670, 4.512	2.93	1.607, 5.361
**Level of education**				
No education	1		1	
Primary	1.16	0.929, 1.451	1.13	0.883, 1.442
Secondary	2.30	1.548, 3.404	2.71	1.737, 4.235
Tertiary	0.96	0.427, 2.158	0.83	0.354, 1.968
**Marital Status**				
Married	1		1	
Not married	1.68	1.168, 2.410	2.31	1.552, 3.444
**Wealth quintile**				
Poorest	1		1	
Poorer	1.80	1.071, 3.029	1.83	1.075, 3.103
Middle	2.42	1.467, 3.988	2.00	1.193, 3.347
Richer	2.19	1.338, 3.592	1.87	1.121, 3.114
Richest	2.22	1.339, 3.691	1.68	0.981, 2.868
**ANC visits**				
< 4 visits	1		1	
4–7 visits	0.96	0.768, 1.205	1.15	0.910, 1.460
8 and more	1.	1.305, 2.455	2.05	1.467, 2.861
**Parity**				
1 birth	1		1	
2 births	3.28	1.610, 6.671	4.26	2.017, 9.020
≥ 3 births	3.14	1.647, 6.004	3.26	1.560, 6.822
**Gestational age**				
Normal	1		1	
Preterm	2.873	1.576, 5.237	3.40	1.809, 6.400
Post-term	1.688	1.143, 2.493	1.50	0.998, 2.259
**Previous pregnancy loss**				
No	1		1	
Yes	1.91	1.549, 2.258	2.03	1.467, 2.575

cOR = Crude Odds Ratio, aOR = Adjusted Odds Ratio, CI = Confidence Interval

## Discussion

The study determined the prevalence of CS in the study population as 14.6%. Level of education, marital status, maternal age and wealth quintile were socio-demographic characteristics significantly associated with CS delivery. Furthermore, gestational age, number of ANC visits, and parity were obstetric characteristics significantly associated with CS delivery. A unique observation from the study was that a history of previous pregnancy loss was significantly associated with CS delivery.

The prevalence of CS delivery in the current study is higher than the 11. 4% [17] in 2014 and 7% [18] in 2013 reported in Ghana, and it is a little lower than the national estimate as per the Family Health Division of GHS [14]. The current prevalence is within the 10–15% rate for regions across the globe recommended earlier by WHO [8]. Furthermore, the CS delivery prevalence in this study was higher than that for Africa (9.2%) and Sub Saharan Africa (5%) [4].

The study found a significant association between CS delivery and socio-demographic determinants of maternal age, level of education, marital status, wealth, and obstetric characteristics such as the number of ANC visits, parity, and gestational age. These findings are consistent with studies in Ghana by Dankwah et al., Apanga and Awoonor-Williams, and Manyeh et al. [17, 19, 20]. In the current study, previous pregnancy loss was positively associated with CS delivery.

A statistically significant association between CS delivery and maternal age was observed in this study. Women above 25 years of age were more likely to deliver by CS as compared to younger women. The finding is consistent with those of Begum et al., Dankwah et al. and Nazir [10, 17, 20]. Ageing and its associated physiological and anatomical changes expose older mothers to high-risk pregnancy complications at delivery. Such women perceive CS as a safer option than vaginal delivery in protecting the fetus [17]. Yassin, on the contrary, observed that younger (30 years and below) women were more likely to have a CS delivery [21].

As observed in this study, women with secondary education were more likely to give birth by CS than those with low or no education, as observed in other studies [7, 17–19, 22]. Mostafa attributed this phenomenon to women being empowered through education – they have autonomy, enhanced decision-making capacity, access to reproductive health care and a deeper understanding of the importance of CS interventions if needed [23]. Khan et al. describe the opposite scenario for the less educated woman, some of whom might perceive delivery by CS as a sign of weakness [22]. Yassin, however, observed no significant association between CS and educational level [21].

Though Rebelo et al. observed that marital status was associated with CS delivery, they did not establish its direction [24]. Unmarried women in the current study were more likely to give birth by CS as compared to their married counterparts. This finding could be attributed to the lack of spousal support during pregnancy or emotional issues they may have experienced due to norms of society, cultural issues, religious affiliation or pressure. These factors require further investigations to explore their influence on CS delivery.

Studies have reported the likelihood of CS delivery to increase with higher wealth quintiles, in congruity with the findings of this study [17, 20, 25, 26]. Most women now do not want to go through vaginal delivery since it is perceived as painful; hence, they opt for CS to avoid the pain [27].

In tandem with the findings of studies by Abbas et al., Apanga and Awoonor-Williams and Nazir, ANC visits increase a woman’s risk of giving birth by CS [19, 20, 28]. Nazir noted that women who receive more ANC were at a higher risk of delivering by CS [20]. Higher ANC visits increase a woman’s chances of delivering by CS, which is in accordance with findings from this study [19, 28]. The explanation for this might be that high-risk pregnancies are identified during ANC visits; therefore, pregnant women in such conditions opt for CS, or physicians provide CS as an intervention to ensure the safety of the mother and baby.

Women in the current study with two or more births were more likely to deliver by CS compared to those with only one child, which is congruent with other studies in India, Denmark and Jordan [29–31]. However, other studies reported that women with increasing parity are less likely to deliver by CS compared to those with low parity or no child [17, 18]. Mgaya et al. observed that by their fifth delivery (i.e., grand-multipara), women were much more likely to give birth by CS - a finding consistent with this current study [32].

Similar to findings from other studies, the current one observed that women who give birth preterm were more likely to deliver by CS than those who give birth at term [1, 7]. The current study also determined a significant association between post-term delivery and CS. Women who deliver post-term were more likely to deliver by CS than term, in accordance with studies from Rénes et al., and Prah et al. [1, 7] who observed an increase in CS in post-term deliveries.

Prior studies have found that women with a history of CS are more likely to have pregnancy loss, miscarriage and stillbirth [33, 34]. On the other hand, the current study found a reverse association between CS and pregnancy loss. The study reveals that women with a history of previous pregnancy loss are more likely to deliver by CS. Evidence from this study and that of O’Neill et al. raise a concern about the bidirectional association between CS delivery and history of previous pregnancy loss.

## Conclusions

The prevalence of CS delivery in the study population falls within the observed GHS and WHO-recommended ranges. Socio-demographic factors such as maternal age, level of education, marital status, and wealth quintile and obstetric factors such as the number of ANC visits, parity and gestational age were significantly associated with CS delivery. History of previous pregnancy loss was additionally identified as significantly associated with CS deliveries.

The Ghana Health Service/Ministry of Health should develop policies that address identified modifiable sociodemographic and obstetric factors to stem the rise in CS deliveries.

## Data Availability

Data used in this study is not publicly available. The data used in the study can be obtained from the corresponding author upon reasonable request.

## Abbreviations

AIC: Akaike’s Information Criteria
ANC: Antenatal Care
aOR: Adjusted Odds Ratio
CI: Confidence Interval
cOR: Crude Odds Ratio
CS: Caesarean Section
EN-INDEPTH: the Every Newborn–International Network for the Demographic Evaluation of Populations and their Health
FBH+: Full Birth History
FPH: Full Pregnancy History
GHS: Ghana Health Service
KHDSS: Kintampo Health and Demographic Surveillance System
KHRC: Kintampo Health Research Centre
WHO: World Health Organization
